# Focal Dermal Hypoplasia Associated With Lymphedema: A Case Report From Saudi Arabia

**DOI:** 10.7759/cureus.37661

**Published:** 2023-04-16

**Authors:** Nouf F Bin Rubaian, Nada Alghamdi, Bayan Alhaddad, Hawra AlJanobi, Abdulmajeed S Alharbi

**Affiliations:** 1 Department of Dermatology, College of Medicine, Imam Abdulrahman Bin Faisal University, Dammam, SAU; 2 Department of Dermatology, King Fahad University Hospital, Dammam, SAU; 3 Biomedical Dental Science Department, College of Dentistry, Imam Abdulrahman Bin Faisal University, Dammam, SAU; 4 Department of Oral and Maxillofacial Pathology, Biomedical Dental Science Department, College of Dentistry, Imam Abdulrahman Bin Faisal University, Dammam, SAU; 5 Medicine, College of Medicine, Imam Abdulrahman Bin Faisal University, Dammam, SAU

**Keywords:** lymphedema, goltz syndrome and blashko lines, dermal atrophy, fdh, focal dermal hypoplasia

## Abstract

Focal dermal hypoplasia (FDH), also known as Goltz syndrome, is a rare syndrome described in the literature. Patchy skin hypoplasia is the most evident sign. Hyperpigmentation, hypopigmentation, papillomas, limb defects, and orofacial manifestations have also been reported. A 12-year-old Saudi girl with unremarkable family history presented with FDH. The diagnosis was confirmed using a genetic study. Physical examination revealed asymmetrical streaks of vermiculate dermal atrophy, telangiectasia with hyperpigmentation, and hypopigmentation on the left half of the face, trunk, and bilateral extremities. It appears along Blashko lines. No mental impairment was observed. Intraoral examination generalized plaque-induced gingivitis with erythematous gingival hyperplasia. Examination of the teeth showed generalized enamel hypoplasia with abnormal tooth formations, malalignment, microdontia, spacing and tilting, and minimal caries. As reported cases of FDH are rare worldwide, this syndrome is yet to be fully understood. As the manifestation of the syndrome varies among cases, the management of each case is unique. This emphasizes the importance of reporting cases of FDH.

## Introduction

Focal dermal hypoplasia, also known as Goltz syndrome, is a rare syndrome that has only been described in approximately 250 individuals in the literature. It is characterized by multiple abnormalities of ectodermal and mesodermal origins. It is brought on by heterozygous and mosaic mutations in the gene called PORCN found in the X chromosome, which is inherited as an X-linked dominant condition. It is well known that PORCN targets Wnt signaling proteins, which are important regulators of skin development in the embryo, as well as bones and other structures. Therefore, this disorder primarily affects women and is lethal in men [1-5].

FDH is characterized by patchy skin hypoplasia, which manifests pathologically as a hypoplastic dermis with thin, sparse collagen bundles and regions of partial to total replacement of dermal connective tissue by adipose tissue, resulting in yellowish herniations. Another sign is hyperpigmentation and hypopigmentation in a blaschkolinear distribution, papilloma, and sparse hair. The condition is also associated with limb defects and ocular malformations, with typical facial dysmorphic features [1-4].

Few cases were mentioned in the literature on the association of Goltz syndrome and lymphedema. Lymphedema is caused by lymph collection due to either primary, which is caused by genetic mutations that impair how the lymphatic system develops, or secondary, brought on by issues with the fluid's circulation and outflow in the lymphatic system or by injury to the lymphatic system itself [6,7]. The lymphatic system is derived from the mesoderm and then gets connected to the venous system at the sixth week of gestation [5-7].

Orofacial manifestations are present in most cases of FDH. Skeletal abnormalities include cleft palate, high arched palate, prognathism, micrognathia, and prominent or pointed chins [4,7,8]. Dental manifestations have been reported in more than half of FDH cases [9], such as hypodontia or oligodontia, microdontia, supernumerary teeth, malocclusion, spacing, taurodontism, gemination, fusion, grooving of the teeth, and deficiency in quantity and/or quality of the enamel and the dentine [4,8-10]. The presence of these manifestations can explain the extensive caries observed in several cases of Goltz syndrome [9]. Tongue hemi-hypoplasia, long-pointed deviated tongue, median cleft of tongue, absence of lingual frenulum, or double lingual frenulum can be observed [8,10,11]. Moreover, oral papilloma is one of the most commonly reported symptoms in the literature [11]. Other oral soft tissue manifestations can be observed, such as gingival hypertrophy and gingivitis, oral lipoma, high or double labial frenulum, and cleft lip [8,9]. The orofacial features vary in each case, but are mostly present (~80%) [12].

The diagnosis of FDH is based on the typical clinical features via clinical examination and imaging based on the abnormality found in the patient and it is confirmed using genetic testing [1-5]. We present the case of a 12-year-old girl with FDH, whose diagnosis was confirmed through genetic testing. She had a heterozygous pathogenic type of the PORCN gene, which resulted in the typical features of FDH, along with congenital absence of the lymphatic system of the left lower extremity and several dental manifestations.

## Case presentation

A 12-year-old Saudi girl with an unremarkable family history, born to consanguineous parents (second-degree cousins), presented with FDH. The mother's age at the time of delivery was 32 years and the father’s age was 33 years. The diagnosis was confirmed through a genetic study that showed a heterozygous splicing likely pathogenic type (class 2) of PORCN gene consistent with X-linked dominant FDH. Genetic sequencing including next-generation sequencing (NGS)-based copy number variation (CNV) analysis. Parents were not sequenced. The mother’s pregnancy was uneventful, and our patient was delivered full-term through normal spontaneous vaginal delivery. The patient was the fifth child and the only one affected among six siblings. The patient’s parents provided written consent for the use of extra- and intraoral photographs and imaging studies for publication. Medical history revealed that the patient had corrective digit syndactyly of the left hand between the second and third fingers at the age of three years and between the right second and third, and the right fourth and fifth toes at the age of nine years. The patient underwent adenoidectomy and tonsillectomy at eight years of age as she had recurrent febrile illness, sore throat, and difficulty in swallowing and sleeping.

**Figure 1:**
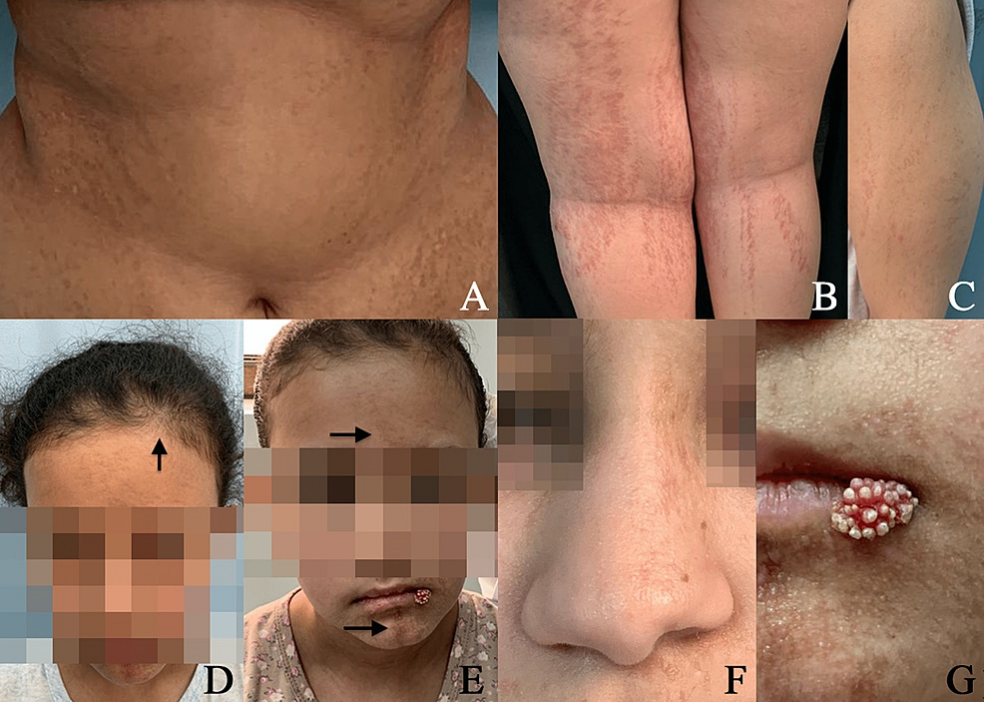
(A,B,C) Streaks of vermiculate dermal atrophy. (D) Decreased thin frontal hair (E) Left half of the face showing multiple liner hyperpigmentation, atrophy and erythema (F) Broad nasal tip and left notched hypoplastic ala. (G) Raspberry papule

Physical examination revealed asymmetrical streaks of vermiculate dermal atrophy, telangiectasia with hyperpigmentation, and hypopigmentation on the left half of the face, the trunk, and the extremities (Figure 1A-1D). Frontal hair density decreased, and generally the hair was thin and frizzy (Figure 1D). The patient showed some dysmorphic facial features with alopecia of the middle third of the left eyebrow and skin atrophy (Figure 1E), a broad nasal tip, and left notched hypoplastic ala nasi (Figure 1F). The patient had a raspberry papule on the left corner of the mouth (Figure 1G). Eye examination revealed left-flick exotropia.

**Figure 2:**
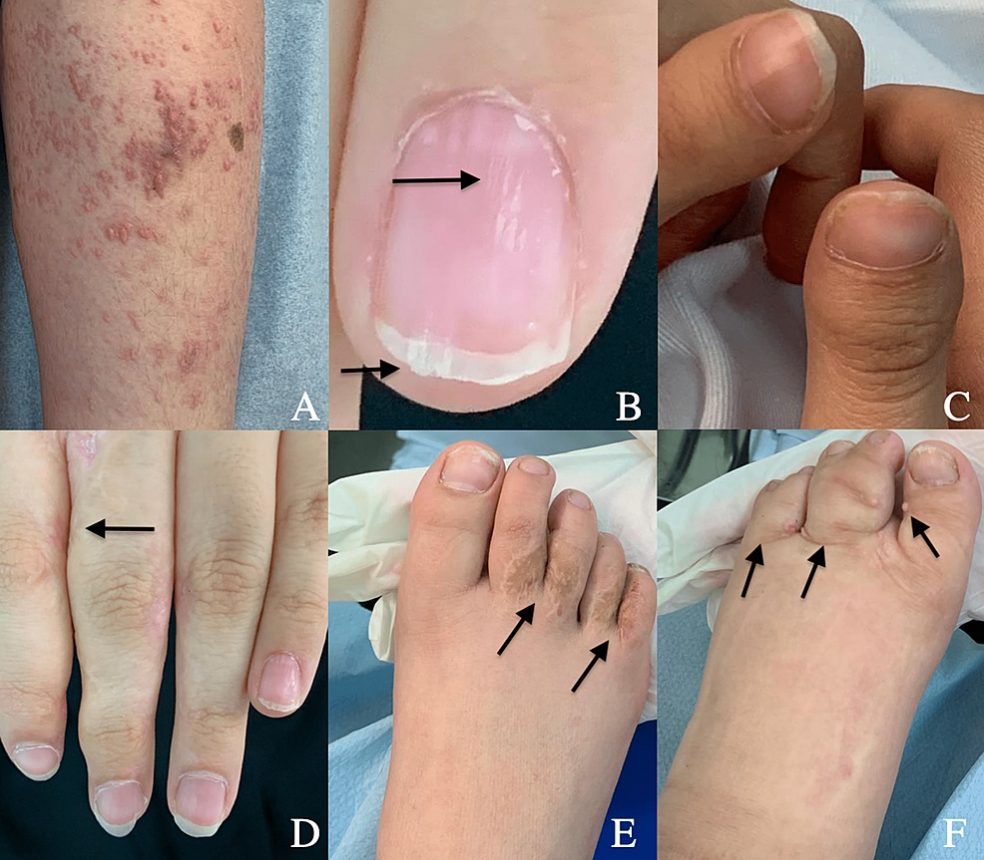
Manifestations in the lower and upper extremities.

On the legs, there were pink papules on top of linear poikilodermatous lesions, representing skin atrophy and fat herniation (Figure 2A). The patient’s nails showed longitudinal ridges, V-notching, and brachyonychia (Figure 2B, 2C). Her hands and feet showed scars at the site of syndactyly correction on the left hand between the second and third fingers at the age of three years (Figure 2D) and on the right feet between the second and third and between the fourth and fifth toes (Figure 2E). Syndactyly was not corrected between the left second and third toes, and between the fourth and fifth toes, with multiple skin-colored to pinkish papules that looked like papillomas (Figure 2F). She also had a congenital left leg swelling, with a circumference of 42 cm, measured 6 cm below the knee; the right leg circumference was 34 cm.

**Figure 3:**
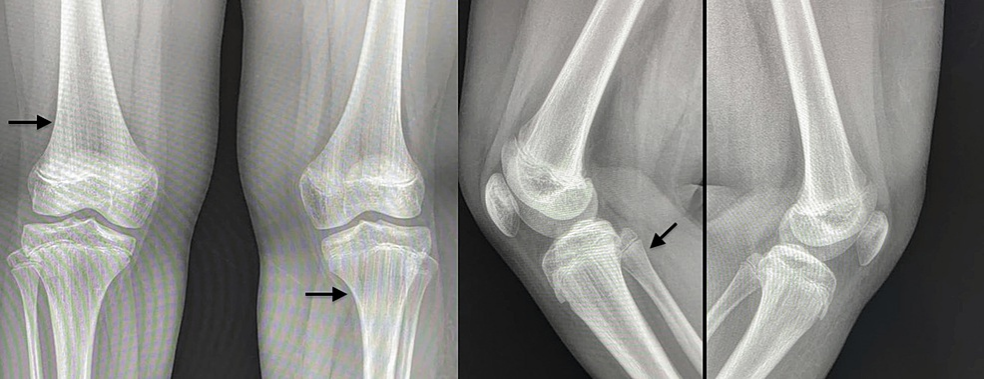
Plain X-ray showed osteopathic striata with prominent vertical striations in metaphysis into diaphysis of femur, tibia and fibula bilaterally

No mental impairment was observed, and the patient used to attend normal school with good results. Imaging and plain radiography showed bilateral osteopathic striata with prominent vertical striations in the metaphysis and extending into the diaphysis of the femur, the tibia, and the fibula (Figure 3). An angiogram of the lower extremities showed a complete absence of the lymphatic system (lymphatic aplasia) in the left lower limb. The type of the angiogram was not mentioned as it was done in another center and the images was not available. Histopathological examination of the skin revealed foci of adipose tissue in the dermis and increased dermal vascularity.

**Figure 4:**
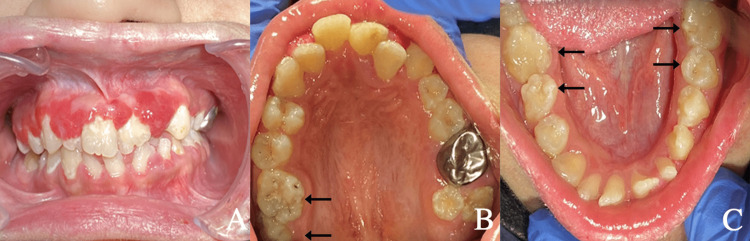
(A) Erythematous gingival hyperplasia (B) Malalignment of maxillary teeth and microdontia of the maxillary left second molar (bold arrow). (C) Abnormal occlusal table of posterior teeth with extra grooving (Arrow).

Upon intraoral examination, the patient had slightly limited mouth opening, with pain in the temporomandibular joint upon opening the mouth with a width of more than two fingers. She had generalized plaque-induced gingivitis with erythematous gingival hyperplasia (strawberry gingivitis) on the maxillary facial side, which was tender to touch (Figure 4A). Teeth examination showed generalized enamel hypoplasia with abnormal tooth formation, malalignment of maxillary teeth, microdontia of the maxillary left second molar, spacing and tilting of the lower anterior teeth, abnormal occlusal table of posterior teeth with extra grooving (mulberry molar looking), and minimal caries (Figure 4B, 4C).

**Figure 5:**
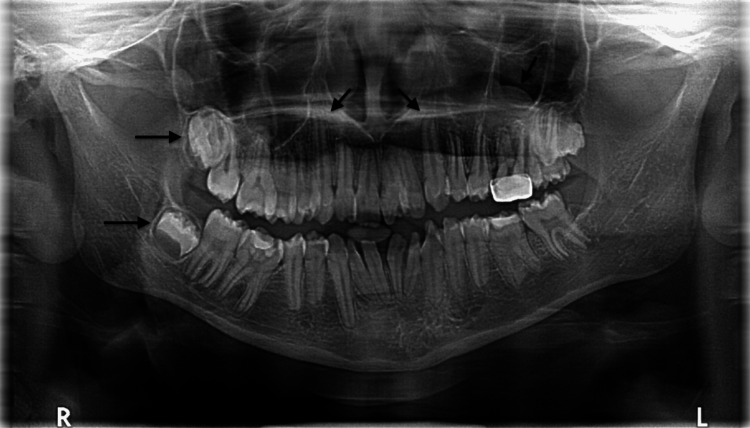
Panoramic radiograph shows impacted maxillary and mandibular right third molar, congenitally missing mandibular left third molar, radiculomegaly of maxillary right canine, dilaceration in maxillary left canine, and maxillary sinus retention cyst on the left side

Panoramic radiographic examination showed an impacted maxillary and mandibular right third molar, congenitally missing mandibular left third molar, radiculomegaly of the maxillary right canine, dilaceration of the maxillary left canine, and maxillary sinus retention cyst on the left side (Figure 5).

## Discussion

Our patient presented with typical Goltz syndrome features with Blaschko line poikiloderma with fat herniation in an asymmetrical pattern, oral papilloma, typical dysmorphic faces, and skeletal defects. Kinmonth classified primary lymphedema according to the age at the time of onset as congenital (present from birth), precox (developing during adolescence), and the tarda type (that occurs after the age of 35 years) [1]; and according to lymphatic vessel abnormalities such as aplasia, hyperplasia, hypoplasia and lymph node fibrosis [2]. Peripheral vessels are either missing or significantly hypoplastic in terms of quantity and size in instances of aplasia/hypoplasia [5]. Maas et al. described one adolescent female who developed leg lymphedema at puberty and did not respond to adjuvant treatment and remained static [5,6]. However, lymphedema in our patient was unilateral (left leg) and the mother stated that it was since birth. Cordero et al. published a case of a girl who had left leg swelling at the age of four years that was worsened by a hip fracture. This was similar to our patient which had unilateral lymphedema, however, our patient had it since birth as the mother stated [6].

In agreement with the literature, our patient showed several oral manifestations of the condition. Wright et al. reported that approximately 30% of the reported cases showed gingivitis, which was present in our case [11]. Other dental findings, such as missing teeth, malalignment, spacing, and microdontia were also seen in our patient. Enamel hypoplasia is a major feature, including our patient, and can be a predisposing factor for dental caries [4]. It has been reported that the severity of the syndrome corresponds to the degree of dental hypoplasia. Mild cases of Goltz syndrome can have normal dentitions [11]. Our case had minimal carious lesions. It was suggested that regular dental care, fissure sealants, and diet counseling will minimize the risk of extensive caries [12].

## Conclusions

In conclusion, as reported cases of FDH are rare worldwide, this syndrome is yet to be fully understood. The manifestation of this syndrome varies among cases which can include defects of the dental structures, skeleton, soft tissues, eyes and skin, emphasizing the importance of reporting these cases. It is recommended to closely monitor patients with FDH to treat them using a transdisciplinary approach and to prevent any complications via close follow-up visits to the dermatologist, frequent eye exams, monitoring of the development and routine screening for behavioral, adaptive and emotional issues.
